# Global research trends of TCM-based nonpharmacologic interventions in breast cancer supportive care: a bibliometric analysis (2000–2025)

**DOI:** 10.3389/fonc.2026.1805786

**Published:** 2026-06-10

**Authors:** Qi Wang, Yun Zhu, Huizhen Feng, Qixia Yu, Changying Ding

**Affiliations:** Department of Breast Surgery, Jiaxing Hospital of Traditional Chinese Medicine, Jiaxing, China

**Keywords:** acupuncture, bibliometric analysis, breast cancer, integrative oncology, non-pharmacological intervention, Qigong, Tai Chi, traditional Chinese medicine

## Abstract

**Background:**

With improving survival in breast cancer, supportive care has increasingly focused on long-term, treatment-related symptom clusters (e.g., pain, fatigue, vasomotor symptoms, insomnia, and anxiety) that impair quality of life and adherence. Within integrative oncology, non-pharmacological Traditional Chinese Medicine (TCM) therapies—especially acupuncture and mind–body exercises (Tai Chi/Qigong)—have attracted growing attention, yet the field’s global structure, collaboration patterns, and evolving research priorities have not been comprehensively mapped.

**Methods:**

A bibliometric visualization study was conducted for English-language articles and reviews published from 2000 to 2025. Records were retrieved on January 8, 2026, from Web of Science Core Collection (WoSCC) and Scopus and deduplicated to form Dataset A for productivity, collaboration, and keyword analyses; Dataset B (WoSCC only) was used for co-citation analyses to ensure consistent cited-reference indexing. PubMed was used as an external source to validate annual publication trends. Analyses were performed using VOSviewer and R-bibliometrix (Biblioshiny).

**Results:**

A total of 1,761 documents from 590 sources were included, showing rapid growth with acceleration after the mid-2010s (10 papers in 2000 and 171 in 2025), and trends were consistent with PubMed (*Spearman’s ρ = 0.982, p = 9.59×10^-19^*). The field displayed a US–China bipolar pattern, with the United States leading in output, citations, and collaboration, and China showing fast recent growth but limited cross-regional institutional linkage. Integrative Cancer Therapies and Supportive Care in Cancer were the main outlets, and the knowledge base centered on RCTs and guidelines, particularly for acupuncture. Keywords revealed a shift from early treatment side-effect management to broader survivorship outcomes, and most recently toward evidence synthesis/implementation and targeted concerns such as sleep disturbance/insomnia, with emerging interest in mechanisms and subtype-related topics.

**Conclusion:**

Research on non-pharmacological TCM therapies for breast cancer has grown rapidly, shifting from “CAM” toward integrative supportive care. Acupuncture and mind–body exercises remain central, and emerging priorities include standardization, implementation, and mechanism-/subtype-informed approaches. Stronger international collaboration and harmonized outcomes are needed to support scalable clinical integration.

## Introduction

Breast cancer remains the most prevalent malignancy among women globally, with its incidence continuing a steady annual climb (1). Significant improvements in survival rates over recent decades are largely attributable to advances in early screening and the refinement of multimodal treatment regimens, including surgery, chemotherapy, radiotherapy, and endocrine therapy (2). However, as survivorship extends, patients frequently encounter a debilitating cluster of treatment-related sequelae, often categorized as “psychoneurological symptoms.” This cluster encompasses aromatase inhibitor-associated musculoskeletal symptoms (AIMSS), chemotherapy-induced nausea and vomiting (CINV), cancer-related fatigue (CRF), lymphedema, insomnia, and anxiety (3, 4). These chronic conditions not only diminish quality of life but also risk compromising adherence to essential therapies, potentially undermining long-term survival outcomes (5, 6).

While pharmacological management remains the standard of care, its efficacy is often constrained by suboptimal tolerability. Common interventions, such as opioids and antidepressants, frequently involve dose-limiting toxicities, risks of dependency, and the clinical complexity of polypharmacy (7). These limitations have catalyzed a paradigm shift toward non-pharmacological alternatives. Non-drug Traditional Chinese Medicine (TCM) therapies—including acupuncture, Tai Chi, Qigong, Tuina, and moxibustion—have gained international recognition for their holistic approach and favorable safety profiles (8). This shift is further supported by recent high-quality randomized controlled trials (RCTs). Furthermore, recent evidence-based guidelines from the Society for Integrative Oncology (SIO) and ASCO recommend selected integrative modalities for specific symptom indications in breast cancer care. For example, the SIO breast cancer guideline (9) supports mind–body approaches (e.g., meditation and relaxation) for anxiety/mood outcomes and supports acupuncture/acupressure for chemotherapy-induced nausea and vomiting, while evidence grades for acupuncture vary across indications. More recently, the SIO–ASCO guideline on integrative medicine for pain management provides updated recommendations for cancer- and treatment-related pain, including aromatase inhibitor–related joint pain in breast cancer (9–11).

Despite the rapid expansion of literature in this field, the sheer volume of data poses a significant challenge for researchers seeking a comprehensive understanding of the academic landscape. Existing systematic reviews and meta-analyses have primarily focused on synthesizing clinical evidence for specific TCM modalities (12). While these reviews provide high-quality evidence for individual interventions, they are inherently limited in their ability to visualize the evolutionary trajectory, collaborative architecture, and emerging intellectual frontiers of the entire domain. Traditional evaluative methods, typically centered on discrete treatments or symptoms, often fail to capture the macroscopic shifts in research hotspots, the topological structure of international collaboration networks, and the transformation of the intellectual base over the past quarter-century.

To address this gap, we conducted a bibliometric and visualization study of English-language articles and review articles published from 2000 to 2025. Using VOSviewer and the R package bibliometrix, we constructed two complementary datasets: a merged corpus from WoSCC and Scopus (Dataset A) for productivity, collaboration, and keyword-based thematic analyses, and a WoSCC-only dataset (Dataset B) for co-citation analyses to ensure consistent cited-reference indexing. PubMed was used as an external validation source to verify annual publication trends. Our objectives were to: (a) map the spatiotemporal distribution of global contributors (countries, institutions, and authors); (b) identify influential journals and seminal references shaping the knowledge base; and (c) track keyword evolution to reveal shifting clinical priorities and emerging mechanistic directions. These findings aim to support clinicians and researchers by providing a panoramic view of non-pharmacological TCM therapies in breast cancer care and by highlighting future opportunities for rigorous trials and precision-oriented symptom management.

## Methods

### Data sources and search strategy

A systematic literature search was conducted on January 8, 2026, across the Web of Science Core Collection (WoSCC), Scopus, and PubMed databases to ensure exhaustive coverage of the research domain. To mitigate potential biases stemming from daily database updates, all bibliographic data acquisition was completed on this single day. For the primary bibliometric analysis, WoSCC (including SCI-Expanded, SSCI, and ESCI) and Scopus were selected as the core data sources, with an emphasis on SCI-Expanded for its comprehensive citation metrics (13). PubMed served as an independent validation database to cross-verify the identified publication trends. For WoSCC and Scopus, the search strategy was developed by combining specific keywords and broad thematic descriptors to maximize recall. In contrast, the PubMed search utilized a rigorous combination of Medical Subject Headings (MeSH) and free-text terms. All strategies focused on two core domains: breast cancer and TCM-based non-pharmacological interventions, such as acupuncture, moxibustion, Tuina, Tai Chi, and Qigong. To ensure thematic precision, terms related to dietary stimulants including green tea, coffee, and caffeine were explicitly excluded. The search period was strictly restricted to 2000–2025, and only original articles and reviews published in English were considered. The formalized search strings for each database are documented in doc S1.

### Datasets used for different analyses

To ensure both broad coverage and consistent citation-linking, we built two datasets for different analyses. Dataset A combined records from WoSCC and Scopus after deduplication, and it was used for publication output, country/institution/author productivity, co-authorship collaboration networks, and keyword-based analyses (keyword co-occurrence and trend topics). Dataset B included WoSCC records only, and it was used for co-citation analyses (author, journal, and reference co-citation networks). We used WoSCC only for co-citation because cited-reference formats and citation indexing rules differ across databases. PubMed records were not merged into the main corpus and were used only to validate publication trends.

### Data screening and collection

The initial retrieval yielded 1,293 records from WoSCC and 1,608 from Scopus. After applying filters for document type (original research and reviews), language (English), and publication year (2000–2025), 1,075 records remained from WoSCC and 1,295 from Scopus. Bibliographic metadata, including full records and cited references, were exported in Plain Text format for WoSCC and CSV format for Scopus. Data processing and deduplication were performed using the mergeDbSources function in the bibliometrix R package (version 4.1.2). A total of 604 duplicate records were identified and removed, yielding 1,766 records after de-duplication.

Retracted-publication screening was conducted prior to the final analyses. Retraction status was determined using retraction-related indexing information in WoSCC and PubMed and was further verified against official retraction notices. Records confirmed as retracted were excluded from Dataset A, Dataset B, and the PubMed validation set, and all affected analyses were re-run after exclusion. The complete list of excluded retracted records is provided in **Supplementary Table 1**. To ensure the integrity of the temporal trend analysis, two early-access records indexed with a publication year of 2026 were excluded. After excluding three retracted publications and the two early-access records, the final standardized Dataset A comprised 1,761 unique documents; the final WoSCC-only Dataset B comprised 1,072 documents.

**Figure 1:**
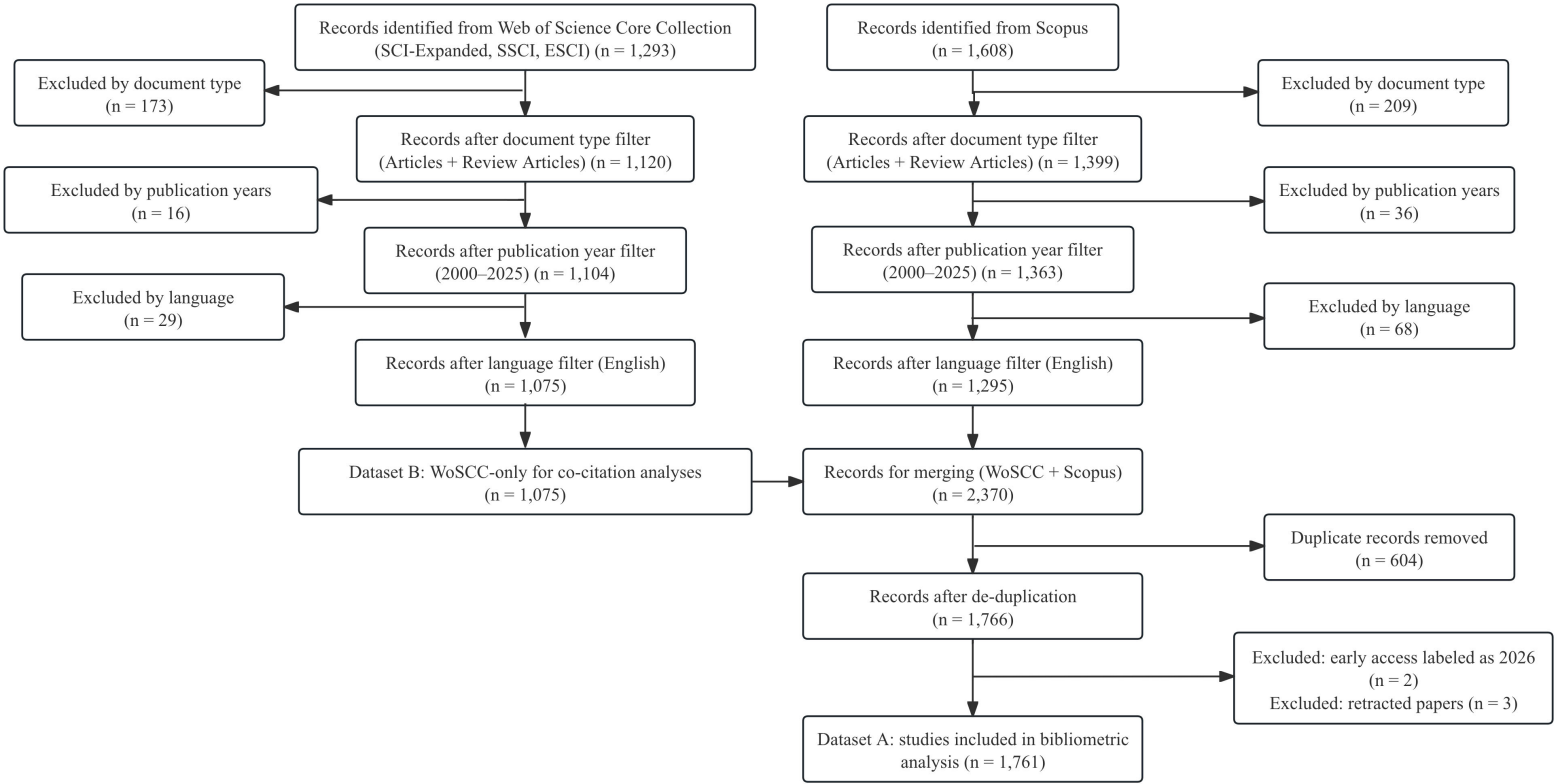
PRISMA flow diagram of the literature selection process.

For external validation, the PubMed search initially yielded 638 records under the same time and language constraints; two retracted records were identified and excluded, resulting in a final PubMed validation set of 636 records. The entire screening process was conducted in strict accordance with the PRISMA (Preferred Reporting Items for Systematic Reviews and Meta-Analyses) (14) guidelines to ensure transparency and reproducibility, as illustrated in **Figure 1**. Prior to analysis, data integrity was rigorously evaluated by verifying key metadata fields, including authors (AU), publication years (PY), affiliations (AU_UN), and author keywords (DE). Missing values in the author keyword field were supplemented by “Keywords Plus” to ensure a comprehensive thematic analysis.

### Bibliometric analysis and visualization

Quantitative analysis and visualization were performed using R-bibliometrix (Biblioshiny), and VOSviewer (version 1.6.20). These tools were employed to analyze publication trends, international collaboration networks among countries and institutions, and author contribution patterns. Thematic evolution was investigated through keyword co-occurrence analysis and co-citation bursts to identify research hotspots and emerging frontiers. To evaluate the reliability of the global trends, a Spearman correlation analysis was conducted to assess the consistency between the primary merged dataset and the PubMed validation records. All statistical tests were two-sided, and a p-value < 0.05 was considered statistically significant.

Concurrently, VOSviewer was employed to construct distance-based, large-scale networks, focusing on spatial collaboration and co-citation patterns. Normalization was performed via the “Association Strength” method, with nodes weighted by citation counts or occurrence frequencies. Minimum frequency thresholds were adjusted iteratively to suppress noise (15). Finally, the biblioshiny interface within the bibliometrix package facilitated the generation of statistical metrics and trend visualizations, including annual growth trajectories and Three-Field Plots (16). Prior to analysis, rigorous data cleaning was conducted to consolidate synonymous terms (e.g., “breast carcinoma” and “breast cancer”). Academic influence was quantified using Journal Impact Factors retrieved from the 2024 Journal Citation Reports (JCR).

It is important to note that geographical attributions (countries and institutions) in this study were strictly based on the authors’ affiliated institutions as indexed in the bibliographic metadata, rather than the physical locations of the clinical treatment centers. For multinational collaborative publications, a standard “full counting” method was applied. Under this approach, a document co-authored by researchers from multiple countries contributes one unit to the publication count of each respective country, and these shared academic affiliations form the edges in the international collaboration networks.

### Ethics

Institutional Review Board (IRB) ethical approval was not required because the data utilized in this study came from the publicly available Web of Science Core Collection database and did not include any interaction with human or animal participants.

## Results

### Overview of data retrieval

A total of 1,761 unique documents from 590 scholarly sources were identified for the period 2000–2025. The dataset exhibited a robust annual growth rate of 12.03%, reflecting an increasing academic interest in integrated Chinese and Western medicine for breast cancer. The research involved 6,280 authors, with an average of 6.19 co-authors per document and an international co-authorship rate of 13.63%. Notably, the average citations per document reached 29.55, and the inclusion of 41,910 cited references provides a solid foundation for further intellectual structure analysis.

### Annual publication and citation trends

**Figure 2:**
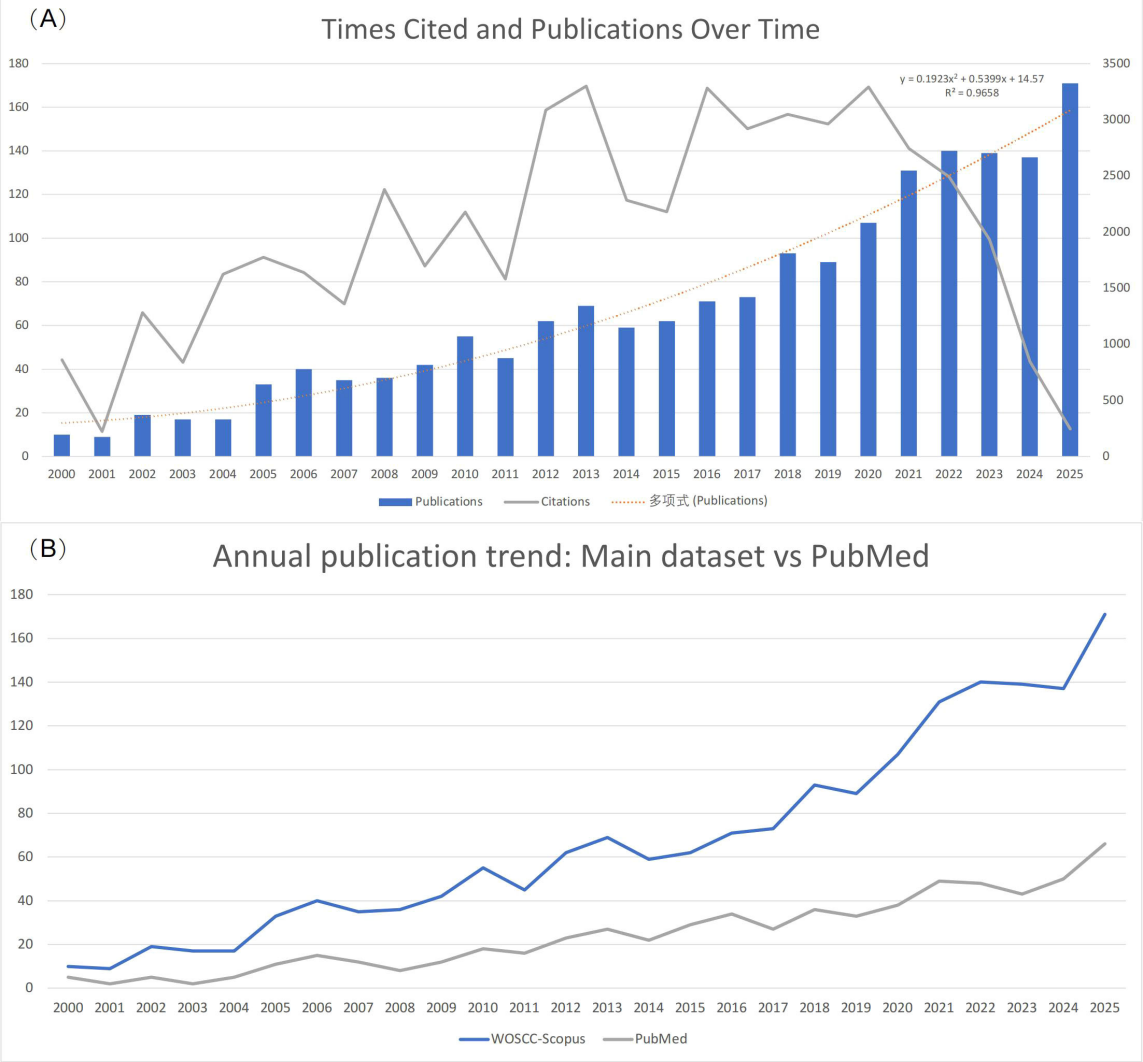
Annual publication and citation trends, and external trend comparison (2000–2025). **(A)** Annual number of publications (bars) and total citations accrued by papers published in each year (line), with a quadratic fit to the publication series. **(B)** Comparison of annual publication trends between the main dataset (WOSCC–Scopus) and PubMed.

As shown in **Figure 2A**, research on non-pharmacological TCM interventions for breast cancer demonstrates a clear long-term growth in annual output with an accelerating trajectory. Annual publications increased from 10 papers in 2000 to 171 papers in 2025 (approximately a 17-fold increase). A quadratic fit to the publication series (*y = 0.1923x^2^ + 0.5399x + 14.57; R² = 0.9658*) suggests that growth has been non-linear and has intensified over time. Citation performance exhibits a different temporal pattern: citations rise for earlier publication cohorts and remain high for papers published roughly between 2012 and 2021, peaking in 2013 (3,298 citations) and remaining above ~2,400 through 2022, followed by a noticeable decline in total citations for papers published in 2024 (847) and 2025 (245). This recent downward trend in citations is a standard bibliometric phenomenon. While early publications have had up to two decades to accumulate citations, recently published papers have not yet existed in the literature long enough to reach their peak citation years. Therefore, the drop post-2020 reflects the time required for new research to be read, assimilated, and formally cited, rather than a decline in the actual quality or impact of recent research.

To benchmark the observed growth pattern against an external database, **Figure 2B** compares annual publication counts from our main dataset with PubMed-derived yearly counts. The two time series show highly consistent year-to-year trends over 2000–2025 (*Spearman’s ρ = 0.982, p = 9.59 × 10^-19^*; 26 yearly observations), supporting the robustness of the temporal trend captured in our dataset. The complete year-by-year counts for both sources are provided in **Supplementary Table 2**.

### Global contribution and cooperation network

**Figure 3:**
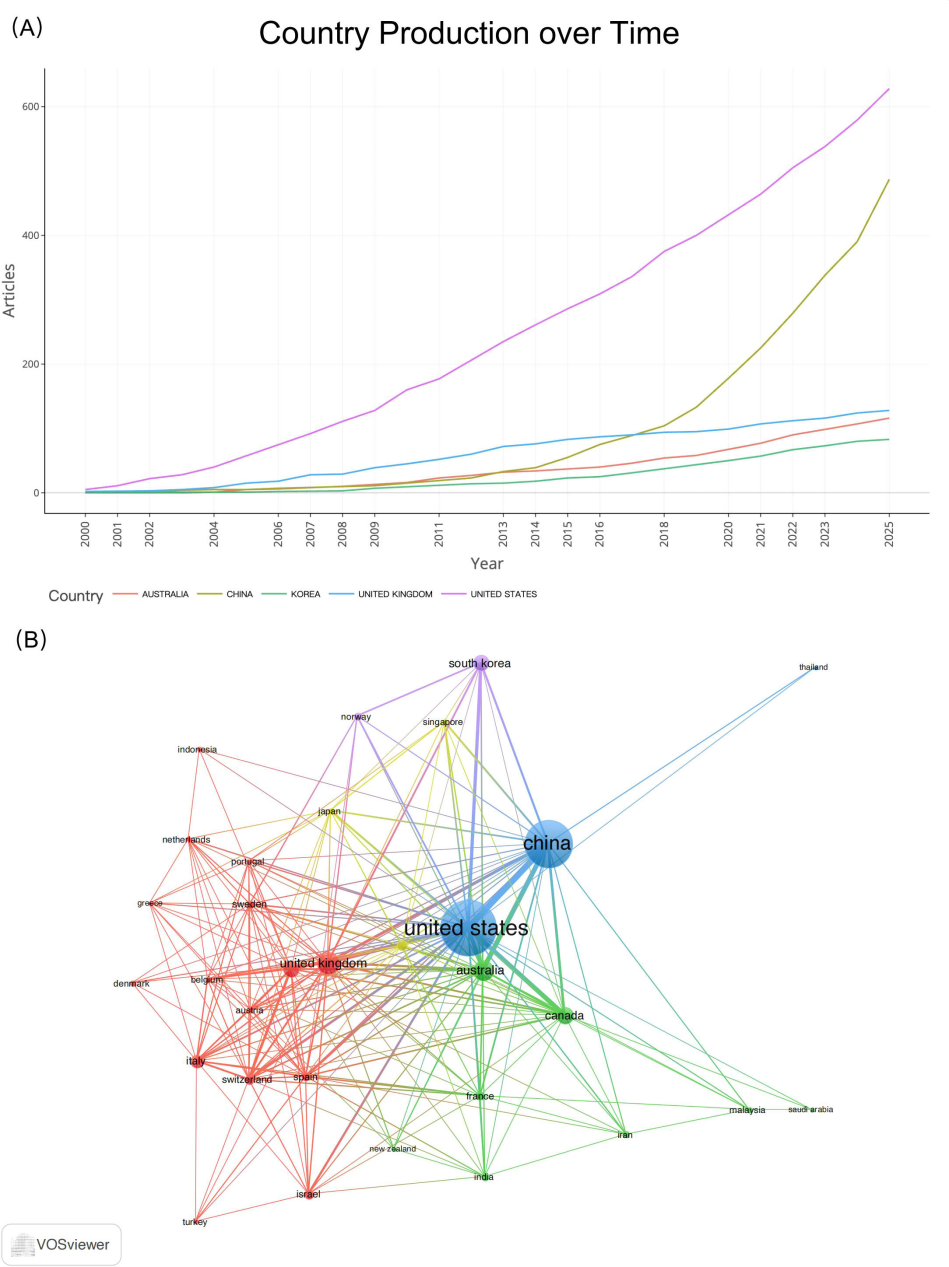
Country production and international collaboration network. **(A)** Country production over time for the leading countries. The y-axis indicates annual publication counts. **(B)** International collaboration network based on country co-authorship. Node size reflects publication volume, link thickness indicates collaboration strength, and colors represent collaboration clusters.

**Table 1** and **Figure 3** demonstrate a research landscape dominated by two major national hubs. Specifically, the United States and China drastically outpace other nations in productivity and collaborative influence. The United States leads the field overall (n = 651). It also has the most citations (27,769) and the highest collaboration strength (TLS = 289). China ranks second (n = 504). It has 9,641 citations and a TLS of 192. The United Kingdom (n = 137; TLS = 106) and Australia (n = 126; TLS = 133) follow. Canada publishes fewer papers (n = 85) but has the highest average citations per item (ACI = 52.61). The United States and the United Kingdom also show high ACI values (42.66 and 43.50). **Figure 3A** shows different growth paths over time. The United States grows steadily across the whole period. China starts later but rises fast in the last decade. The increase becomes sharper after the mid-2010s. The United Kingdom, Australia, and South Korea grow more slowly. **Figure 3B** maps international collaboration. The United States sits at the center of the network. It connects strongly with many countries. China forms a second hub with broad links. The network also shows regional clusters. Several European countries group together, including the United Kingdom, Germany, and Italy. Many Asia–Pacific partners cluster around China. Australia sits between the two hubs. It has strong links with both the United States and China. This suggests a bridging role in global collaboration.

**Table 1:** The top 10 most active countries ranked by publication volume.

Rank	Country	Documents	Citations	ACI	TLS
1	United States	651	27769	42.66	289
2	China	504	9641	19.13	192
3	United Kingdom	137	5959	43.50	106
4	Australia	126	4888	38.79	133
5	Canada	85	4472	52.61	89
6	South Korea	84	1904	22.67	46
7	Germany	83	2329	28.06	71
8	Italy	58	1434	24.72	41
9	Brazil	40	859	21.48	27
10	Israel	34	922	27.12	26

ACI, Average Citations per Item; TLS, Total Link Strength. Rankings were based on the number of documents.

### Institutional collaboration analysis

**Figure 4:**
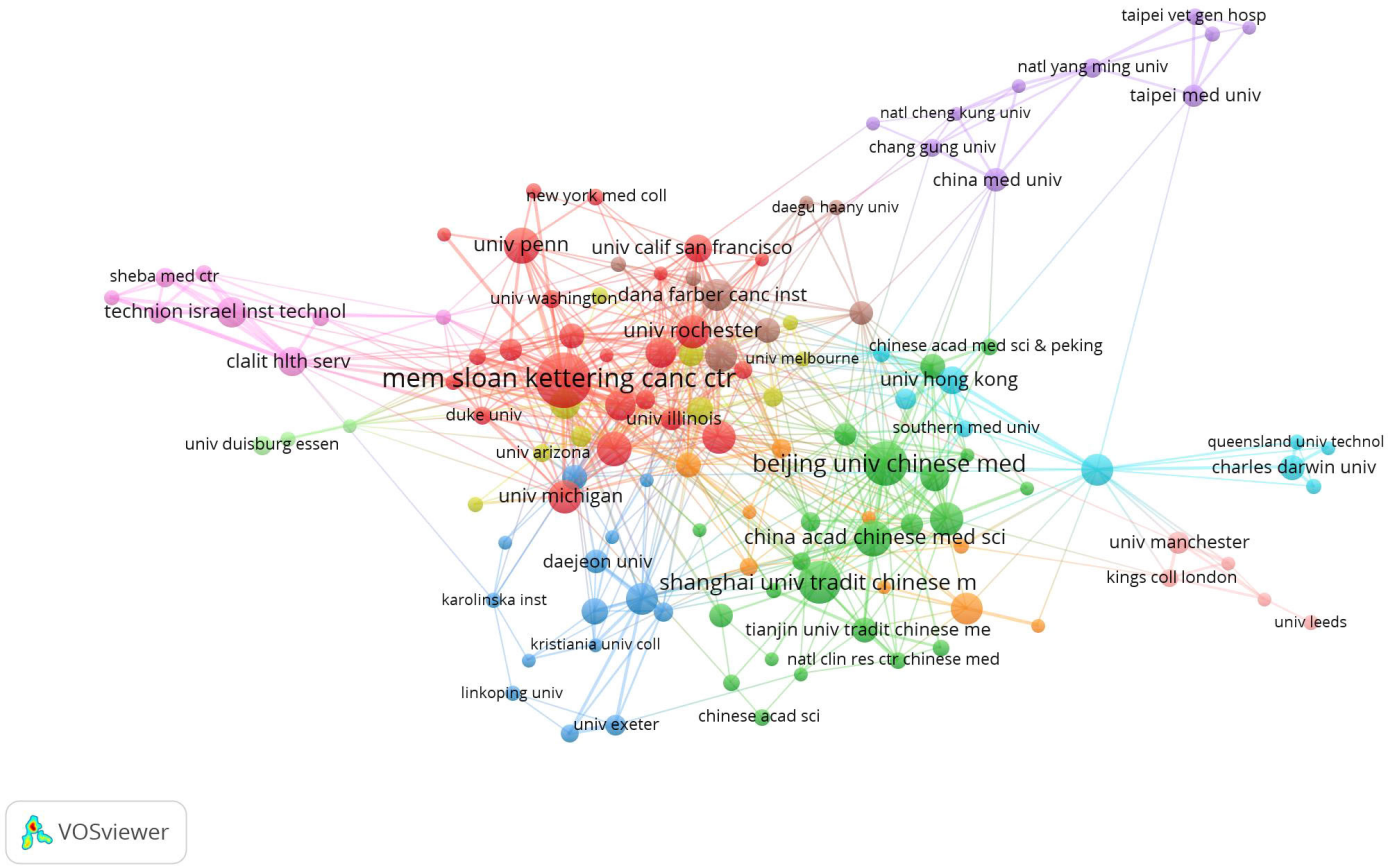
Institutional collaboration network and top productive institutions (generated by VOSviewer).

At the institutional level, the collaboration map (**Figure 4**) shows clear regional clustering. This pattern is consistent with the country-level network. Most links occur within the same region, with fewer strong ties across regions. The 10 most productive institutions are listed in **Table 2**. Memorial Sloan Kettering Cancer Center ranks first (50 documents). It also has the highest total link strength (TLS = 89) and the most citations (2,977). Beijing University of Chinese Medicine (34 documents; TLS = 51) and Shanghai University of Traditional Chinese Medicine (32 documents; TLS = 25) rank next. China Academy of Chinese Medical Sciences also contributes substantially (24 documents; TLS = 39). Among US institutions, the University of Texas MD Anderson Cancer Center shows high impact (23 documents; 2,288 citations). It has the highest average citations per item (ACI = 99.48). The University of California, Los Angeles also shows high impact (ACI = 93.27). The University of Rochester has a similar pattern (ACI = 89.23). **Figure 4** further highlights the structure of institutional collaboration. A large US-centered cluster is anchored by Memorial Sloan Kettering Cancer Center. It includes the University of Texas MD Anderson Cancer Center and the University of Pennsylvania, among others. A second major cluster is centered on mainland China. It is led by Beijing University of Chinese Medicine, Shanghai University of Traditional Chinese Medicine, and China Academy of Chinese Medical Sciences. These institutions show dense internal connections. However, direct links between the two major clusters are less prominent. Several nodes appear to connect sub-networks, including Hong Kong Polytechnic University and the University of California, Los Angeles. Overall, the network suggests strong intra-regional integration but limited inter-regional coupling. This structure supports the need for more direct, multi-center collaboration between leading Chinese TCM institutions and major Western oncology centers.

**Table 2:** The top 10 most active institutions ranked by publication volume.

Rank	Institution	Documents	Citations	ACI	TLS
1	Memorial Sloan Kettering Cancer Center	50	2977	59.54	89
2	Beijing University of Chinese Medicine	34	542	15.94	51
3	Shanghai University of Traditional Chinese Medicine	32	510	15.94	25
4	China Academy of Chinese Medical Sciences	24	611	25.46	39
5	University of Pennsylvania	24	1346	56.08	28
6	University of Texas MD Anderson Cancer Center	23	2288	99.48	47
7	Chengdu University of Traditional Chinese Medicine	22	371	16.86	24
8	University of California, Los Angeles	22	2052	93.27	27
9	University of Rochester	22	1963	89.23	39
10	University of Michigan	21	1214	57.81	43

ACI, Average Citations per Item; TLS, Total Link Strength. Rankings were based on the number of documents.

### Analysis of authors

**Figure 5:**
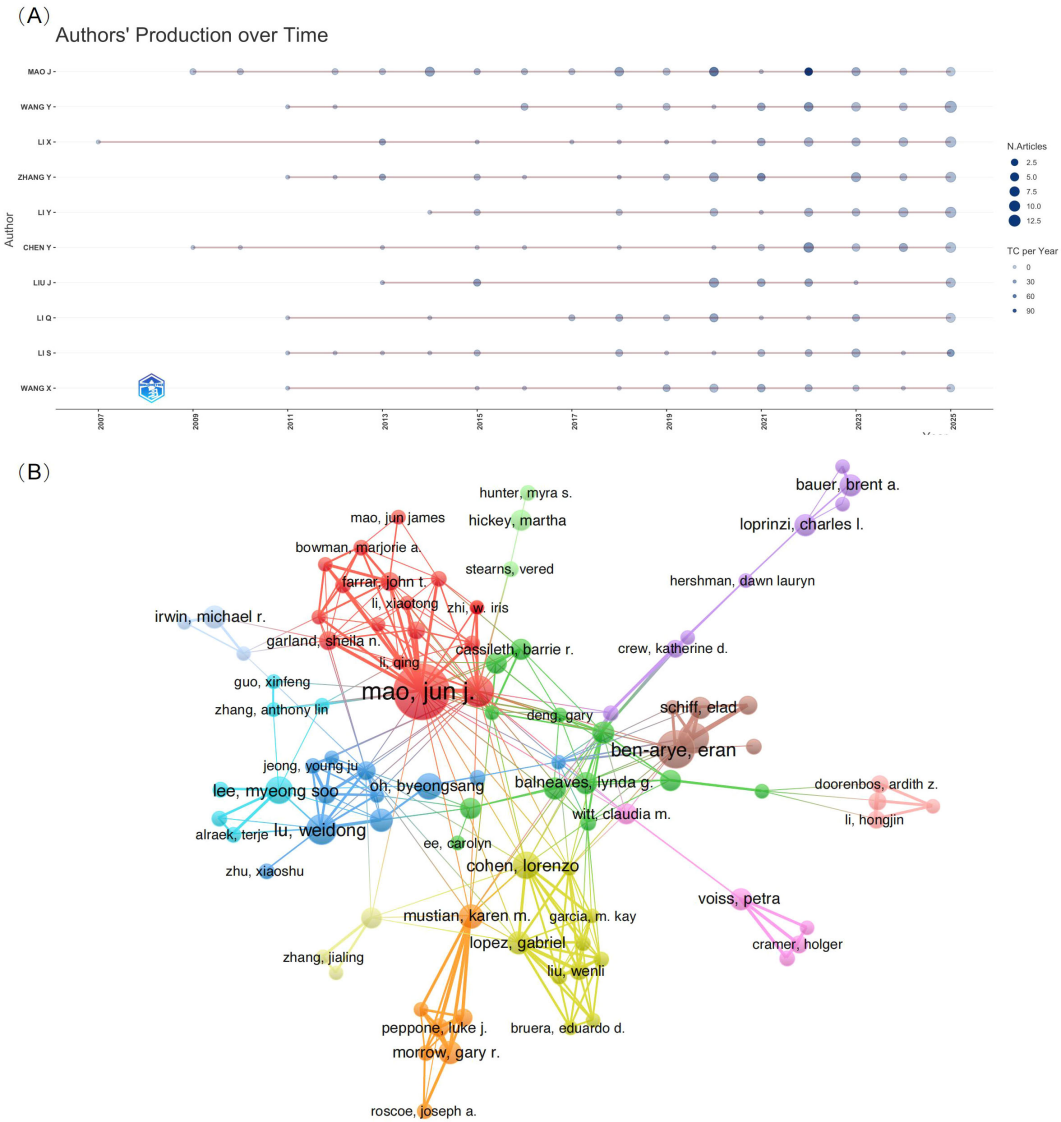
Author productivity and co-authorship network. **(A)** Authors’ production over time. Bubble size indicates the number of articles published in each year, and color intensity reflects citations per year. **(B)** Co-authorship network of authors. Node size represents publication volume, link thickness indicates collaboration strength, and colors denote collaboration clusters.

Identifying pivotal researchers is essential for outlining the field’s development and its active research forces. Based on the authorship analysis (**Table 3**; **Figure 5A**), Mao, Jun J. is the most prolific contributor (45 documents), with the highest overall citation count among the top authors (2,029 citations) and the strongest collaborative connectivity (TLS = 90), highlighting his central role in both productivity and collaboration. He is followed by Ben-Arye, Eran (24 documents; 459 citations; TLS = 51) and Bao, Ting (18 documents; 948 citations; TLS = 47), indicating a stable core group of high-output researchers in this domain. While publication volume reflects productivity, Average Citations per Item (ACI) provides a complementary indicator of per-paper influence. Notably, Irwin, Michael R. shows the highest academic impact among the top authors (ACI = 128.09; 1,409 citations across 11 documents), followed by Mustian, Karen M. (ACI = 101.58; 1,219 citations across 12 documents) and Cohen, Lorenzo (ACI = 89.57; 1,254 citations across 14 documents). These authors demonstrate substantial per-publication visibility, suggesting that their contributions are frequently used as key evidence or conceptual anchors in subsequent studies. The temporal distribution of author productivity (**Figure 5A**) suggests sustained engagement by leading authors—particularly Mao, Jun J.—with continued output across the study period rather than short-lived bursts. From a collaboration perspective (**Figure 5B**), the co-authorship network presents a clear hub-and-cluster structure: Mao, Jun J. occupies a prominent central position, linking multiple author groups, whereas several other influential authors form comparatively tighter subgroups with fewer cross-cluster connections (e.g., lower TLS values for some high-ACI authors such as Irwin, Michael R.). Overall, these patterns indicate that the field has formed a recognizable collaborative core, accompanied by several specialized teams contributing high-impact work.

**Table 3:** Top 10 authors and bibliometric indicators.

Rank	Author	Documents	Citations	ACI	TLS
1	Mao JJ	45	2029	45.09	90
2	Ben-Arye E	24	459	19.13	51
3	Bao T	18	948	52.67	47
4	Lu W	17	338	19.88	32
5	Samuels N	17	192	11.29	40
6	Cohen L	14	1254	89.57	37
7	Lee MS	14	498	35.57	19
8	Oh B	13	650	50.00	7
9	Mustian KM	12	1219	101.58	37
10	Irwin MR	11	1409	128.09	10

ACI, Average Citations per Item; TLS, Total Link Strength. Rankings were based on the number of documents.

### Analysis of journals

**Figure 6:**
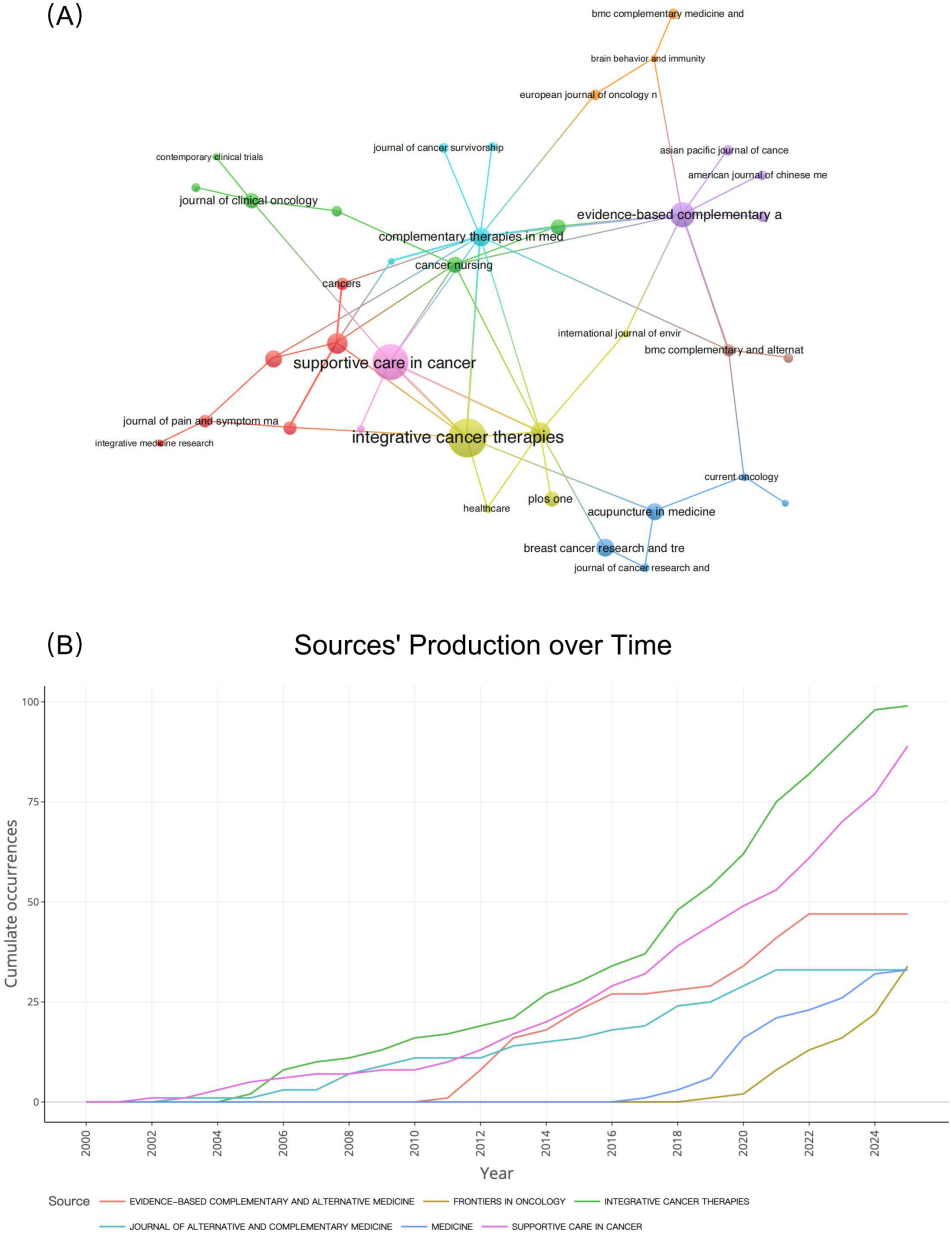
Source network and source production over time. **(A)** journal collaboration network. Node size represents publication volume, link thickness indicates strength of source relatedness, and colors denote clusters. **(B)** Sources’ production over time for the leading journals.

The retrieved literature is distributed across 590 academic journals, indicating a broad but increasingly structured dissemination landscape (**Table 4**; **Figure 6**). As shown in the source network (**Figure 6A**), Integrative Cancer Therapies occupies a central position and serves as the leading publication venue (99 documents, IF = 2.8, Q2), followed closely by Supportive Care in Cancer (89 documents, IF = 3.0, Q1). These two outlets form the primary communication hubs for research at the intersection of integrative oncology and supportive care, consistent with their high productivity and strong connectivity in the visualization. Beyond the two core sources, several journals contribute substantively to the field’s volume and/or influence. Evidence-Based Complementary and Alternative Medicine ranks third in productivity (47 documents, 1,247 citations), but it was removed from the Web of Science Core Collection in 2023; therefore, current Impact Factor and JCR quartile information are unavailable, although its historical role within the study period remains notable. Additional productive outlets include Frontiers in Oncology (34 documents, IF = 3.3, Q2) and Medicine (33 documents, IF = 1.4, Q2). Importantly, citation-oriented indicators suggest that some journals, despite moderate output, provide high per-article impact—most notably Complementary Therapies in Medicine (30 documents, 1,421 citations, IF = 3.5, Q1; ACI = 47.37) and Breast Cancer Research and Treatment (28 documents, 1,184 citations, IF = 3.0, Q2; ACI = 42.29), reflecting their role in disseminating highly cited integrative and symptom-management evidence. Temporal trends (**Figure 6B**) further demonstrate a clear expansion in specialized outlets after approximately 2016, with sustained growth trajectories for Integrative Cancer Therapies and Supportive Care in Cancer, alongside the emergence and gradual accumulation of publications in journals such as Frontiers in Oncology. Collectively, these patterns suggest that the field has moved from scattered publication across complementary medicine outlets toward more concentrated dissemination in journals explicitly aligned with integrative oncology, supportive care, and quality-of-life–oriented cancer research.

**Table 4:** The top 10 most productive journals.

Rank	Journal	Documents	Citations	Country	Publisher	IF	JCR	ACI	TLS
1	Integrative Cancer Therapies	99	1910	United States	SAGE Publications	2.8	Q2	19.29	10
2	Supportive Care in Cancer	89	2420	Germany	Springer Nature	3	Q1	27.19	9
3	Evidence-Based Complementary and Alternative Medicine *	47	1247	United Kingdom	Wiley	N/A	N/A	26.53	10
4	Frontiers in Oncology	34	253	Switzerland	Frontiers Media SA	3.3	Q2	7.44	8
5	Medicine	33	166	United States	Wolters Kluwer	1.4	Q2	5.03	9
6	Complementary Therapies in Medicine	30	1421	United Kingdom	Elsevier	3.5	Q1	47.37	14
7	Breast Cancer Research and Treatment	28	1184	Netherlands	Springer Nature	3	Q2	42.29	2
8	European Journal of Integrative Medicine	25	97	Germany	Elsevier	1.7	Q3	3.88	3
9	Acupuncture in Medicine	24	522	United Kingdom	SAGE Publications	2.6	Q2	21.75	3
10	Cancer Nursing	22	531	United States	Wolters Kluwer	2.5	Q1	24.14	6

IF, Impact Factor; JCR, Journal Citation Reports Quartile; ACI, Average Citations per Item. Rankings were based on the number of documents.

*Evidence-Based Complementary and Alternative Medicine was discontinued from the Web of Science Core Collection (WoSCC) in 2023; therefore, its 2023/2024 Impact Factor and JCR quartile are not available (N/A). However, its publication data prior to delisting remains included to reflect the historical productivity of the field.

### Analysis of keywords

**Figure 7:**
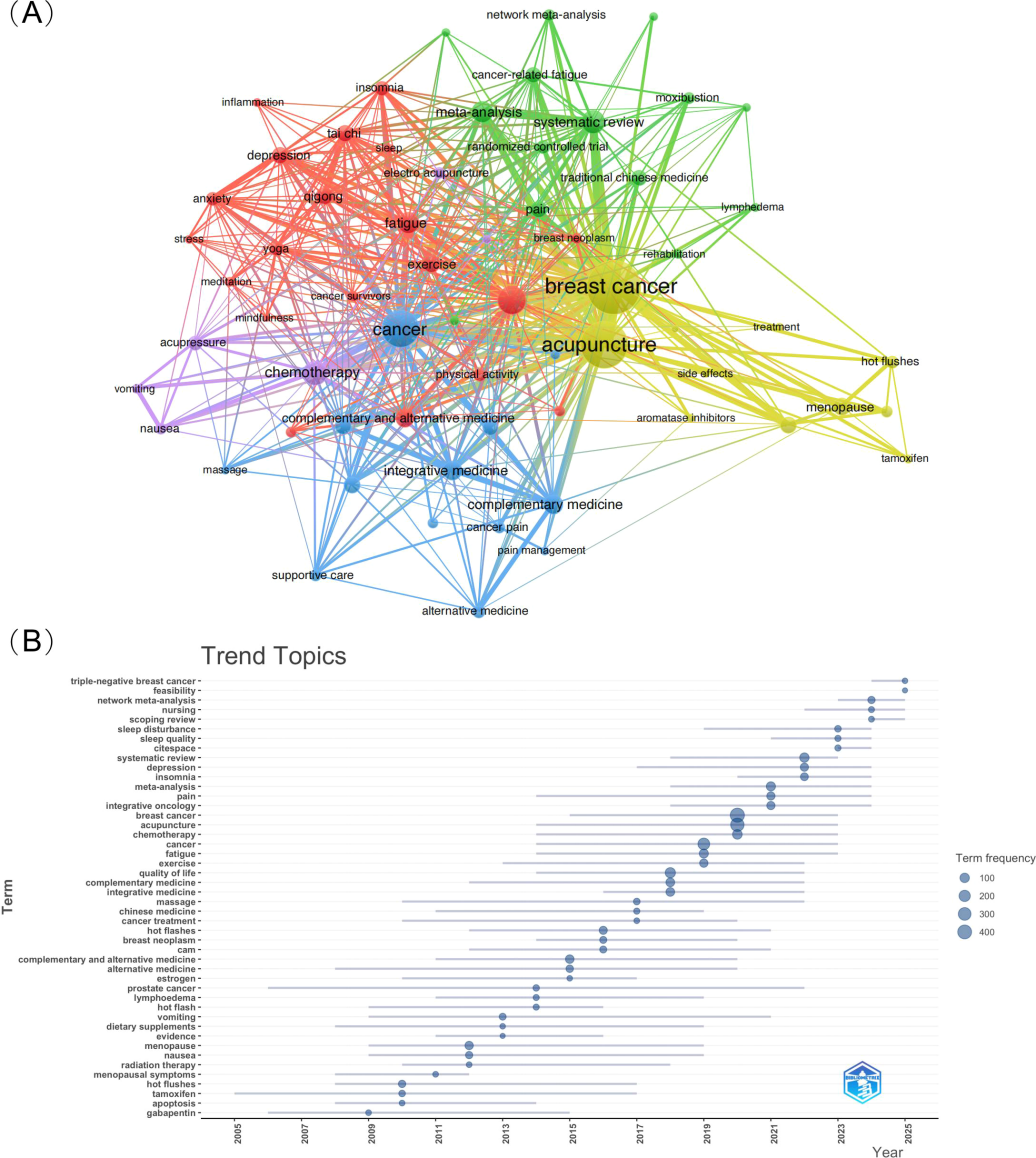
Author keyword network and trend topics. **(A)** Co-occurrence network of author keywords. Node size represents keyword frequency. Links represent co-occurrence relationships, and colors indicate clusters. **(B)** Trend topics over time based on author keywords. Point size indicates term frequency, and the horizontal line shows the time span of each term.

We performed a keyword co-occurrence analysis based on author keywords (**Figure 7A**). The network was dense and was dominated by the hub terms “breast cancer” and “acupuncture.” These keywords were strongly connected with “cancer,” “chemotherapy,” “quality of life,” “pain,” “fatigue,” and “side effects.” This pattern suggests that the literature mainly discusses acupuncture as supportive care for symptom management in breast cancer. The cluster map showed several thematic groups. One cluster focused on hormone therapy and vasomotor symptoms, including “menopause,” “hot flushes,” “tamoxifen,” and “aromatase inhibitors.” A second cluster focused on symptoms and psychosocial issues, including “fatigue,” “insomnia,” “depression,” “anxiety,” and “stress,” and it also included nonpharmacological approaches such as “yoga,” “mindfulness,” “tai chi,” “qigong,” and “exercise.” A third cluster reflected evidence and study design, with terms such as “systematic review,” “meta-analysis,” “network meta-analysis,” and “randomized controlled trial.” It was linked to “traditional Chinese medicine,” “moxibustion,” “rehabilitation,” and “lymphedema.” A fourth cluster represented integrative oncology and supportive care, including “integrative medicine,” “complementary medicine,” “supportive care,” “palliative care,” and “pain management.” A smaller cluster addressed gastrointestinal symptoms, including “nausea,” “vomiting,” and “acupressure.”

To validate the themes from author keywords, we analyzed PubMed MeSH terms for the PubMed subset (N = 636). Among these records, 509 had assigned MeSH terms. **Supplementary Table 3** summarizes the top 20 major-topic MeSH terms and the top 20 MeSH terms overall. The most frequent major-topic terms were Breast Neoplasms (n=393, 77.21%) and Acupuncture Therapy (n=166, 32.61%). Other frequent major-topic terms included Antineoplastic Agents (n=50), Hot Flashes (n=50), Cancer Survivors (n=43), and Tai Ji (n=42). Major-topic terms also covered key modalities and symptom domains, such as Acupressure, Electroacupuncture, and Qigong. Consistent with the hormone-related cluster, Menopause and Aromatase Inhibitors were also among the top major-topic terms. In the analysis of all MeSH terms, common indexing tags were frequent (e.g., Humans and Female). Symptom and outcome terms were also prominent, including Treatment Outcome (n=115), Randomized Controlled Trials as Topic (n=92), Fatigue (n=72), and Hot Flashes (n=67). Overall, the MeSH results were consistent with the keyword network and supported the main thematic structure.

Trend topic analysis (**Figure 7B**) showed an early focus on menopause- and drug-related terms, such as “tamoxifen,” “menopausal symptoms,” and “hot flushes.” Later, attention shifted to broader care concepts, including “integrative medicine,” “complementary and alternative medicine,” and “quality of life.” In recent years, evidence synthesis and implementation topics became more visible, including “systematic review,” “meta-analysis,” “network meta-analysis,” “scoping review,” “feasibility,” and “nursing.” Sleep-related topics also increased, including “sleep quality,” “sleep disturbance,” and “insomnia”.

### Co-citation analysis based on the web of science database

**Figure 8:**
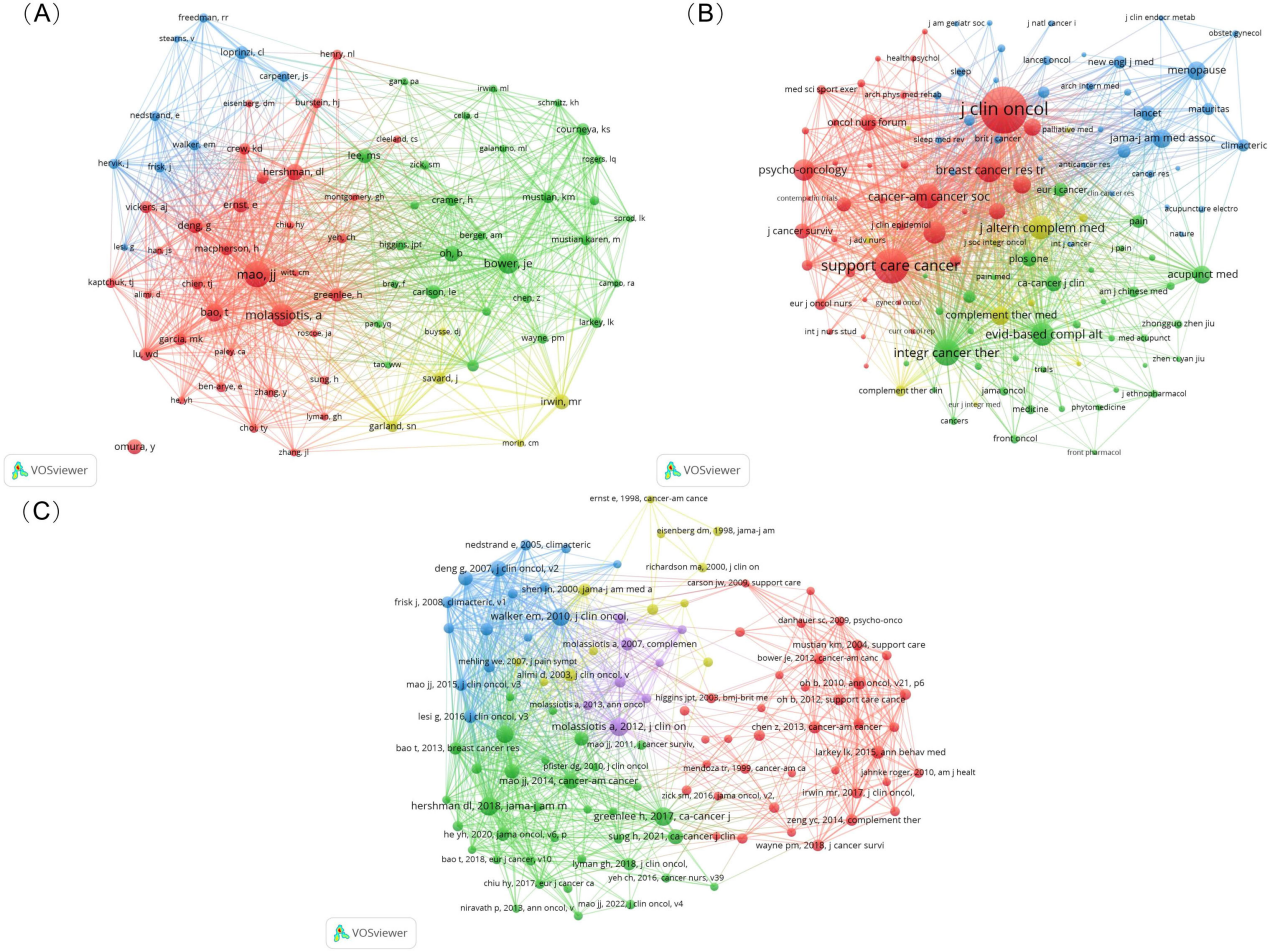
Co-citation analysis based on the Web of Science (WoS) database. **(A)** Author co-citation network of highly co-cited authors. **(B)** Journal co-citation network. **(C)** Reference co-citation network of highly co-cited documents. In all maps, node size indicates co-citation frequency, link thickness represents total link strength (TLS), and colors denote clusters.

All co-citation analyses were conducted using WoS as the sole data source. The co-citation networks reveal a stable and highly connected intellectual structure (**Figures 8A–C**; **Tables 5**, **6**). In the author co-citation network, 78 highly co-cited authors formed several closely linked clusters (**Figure 8A**). Mao, J.J. ranked first in both influence indicators (421 co-citations; TLS = 6388). Molassiotis, A. (331; TLS = 4655) and Bower, J.E. (291; TLS = 4760) followed. The network structure suggests four main knowledge pillars. The first pillar is integrative oncology and acupuncture evidence, represented by Mao, Molassiotis, Bao, Hershman, and Greenlee. The second pillar focuses on exercise and mind–body interventions, with Bower as a core author. The third pillar addresses sleep disturbance and behavioral therapies, centered on Irwin, Savard, and Garland. The fourth pillar relates to symptom control trials and measurement work, represented by Loprinzi and Carpenter. In the journal co-citation network, a threshold of 70 citations per source retained 122 journals from 8,784 cited sources (**Figure 8B**; **Table 5**). The most frequently co-cited journal was Journal of Clinical Oncology (2,956 citations; TLS = 139,717), followed by Supportive Care in Cancer (1,836; TLS = 96758) and Integrative Cancer Therapies (1,152; TLS = 63,899). The journal clusters link oncology and supportive care outlets with integrative medicine and acupuncture-focused journals, indicating sustained cross-field knowledge exchange. The reference co-citation map further shows that the knowledge base is dominated by high-level clinical evidence (**Figure 8C**; **Table 6**). The most frequently cited document was a 2012 pragmatic randomized controlled trial on acupuncture for cancer-related fatigue (17) (n = 97; TLS = 872). It was closely followed by the 2017 SIO clinical practice guideline on evidence-based integrative therapies (9) (n = 93; TLS = 751). Several highly connected core references were clinical trials or randomized controlled trials, and many focused on acupuncture-related interventions for treatment-related symptoms. Key symptom targets included fatigue and aromatase inhibitor–associated arthralgia. Together, these patterns indicate that the field is anchored in evidence-based oncology care while maintaining a strong integrative and symptom-management orientation.

**Table 5:** The top 10 most frequently co-cited journals and co-cited authors.

Rank	Journal	Journal TLS	Journal citations	Publisher	IF	OA	Author	Author TLS	Author citations
1	Journal of Clinical Oncology	139717	2956	Wolters Kluwer	43.4	Hybrid	Mao JJ	6388	421
2	Supportive Care in Cancer	96758	1836	Springer Nature	3.0	Hybrid	Molassiotis A	4655	331
3	Integrative Cancer Therapies	63899	1152	SAGE Publications	2.8	Fully OA	Bower JE	4760	291
4	Cancer	55431	1064	Wiley	5.1	Hybrid	Bao T	3902	227
5	Breast Cancer Research and Treatment	52621	1054	Springer Nature	3.0	Hybrid	Hershman DL	2868	200
6	Journal of Alternative and Complementary Medicine	45185	933	Mary Ann Liebert, Inc.	2.4	Hybrid	Oh B	3650	181
7	Evidence-Based Complementary and Alternative Medicine*	43251	898	Wiley	N/A	Fully OA	Irwin MR	2040	173
8	Journal of Pain and Symptom Management	41544	838	Elsevier	3.5	Hybrid	Deng G	3598	172
9	Psycho-Oncology	48998	791	Wiley	3.5	Hybrid	Ernst E	2223	163
10	JAMA-Journal of the American Medical Association	29400	651	American Medical Association	55.0	Hybrid	Omura Y	20	162

TLS, Total Link Strength. Ranked by citations; IF, Impact Factor; OA, Open Access.

*Evidence-Based Complementary and Alternative Medicine was discontinued from the Web of Science Core Collection (WoSCC) in 2023; therefore, its 2023/2024 Impact Factor and JCR quartile are not available (N/A). However, its publication data prior to delisting remains included to reflect the historical productivity of the field.

**Table 6:** The top 10 most cited co-cited references.

Rank	Co-cited reference title	TLS	Citations	Year	Journal	Type
1	Acupuncture for Cancer-Related Fatigue in Patients With Breast Cancer: A Pragmatic Randomized Controlled Trial (17)	872	97	2012	Journal of Clinical Oncology	Randomized Controlled Trial
2	Clinical practice guidelines on the evidence‐based use of integrative therapies during and after breast cancer treatment (9)	751	93	2017	CA: A Cancer Journal for Clinicians	Practice Guideline
3	Effect of Acupuncture vs Sham Acupuncture or Waitlist Control on Joint Pain Related to Aromatase Inhibitors Among Women with Early-Stage Breast Cancer (3)	805	92	2018	JAMA-Journal of the American Medical Association	Clinical Trial
4	Acupuncture Versus Venlafaxine for the Management of Vasomotor Symptoms in Patients with Hormone Receptor–Positive Breast Cancer: A Randomized Controlled Trial (18)	954	90	2010	Journal of Clinical Oncology	Clinical Trial
5	Randomized, Blinded, Sham-Controlled Trial of Acupuncture for the Management of Aromatase Inhibitor–Associated Joint Symptoms in Women with Early-Stage Breast Cancer (19)	928	82	2010	Journal of Clinical Oncology	Clinical Trial
6	Randomized, Controlled Trial of Acupuncture for the Treatment of Hot Flashes in Breast Cancer Patients (20)	890	75	2007	Journal of Clinical Oncology	Clinical Trial
7	Electroacupuncture for fatigue, sleep, and psychological distress in breast cancer patients with aromatase inhibitor‐related arthralgia: A randomized trial (21)	757	68	2014	Cancer	Clinical Trial
8	Global Cancer Statistics 2020: GLOBOCAN Estimates of Incidence and Mortality Worldwide for 36 Cancers in 185 Countries (22)	451	66	2021	CA: A Cancer Journal for Clinicians	Article
9	Acupuncture for the treatment of hot flashes in breast cancer patients, a randomized, controlled trial (23)	775	65	2009	Breast Cancer Research and Treatment	Clinical Trial
10	A randomized trial of electro-acupuncture for arthralgia related to aromatase inhibitor use (24)	772	62	2014	European Journal of Cancer	Randomized Controlled Trial

TLS, Total Link Strength. Ranked by citations.

## Discussion

### General trends and global disparities

This study delineates the evolving research landscape of non-pharmacological TCM-related interventions in breast cancer management over 2000–2025 using a merged WoSCC–Scopus corpus (Dataset A), co-citation mapping from WoSCC (Dataset B), and PubMed-based trend validation. The longitudinal data reveal a significant paradigm shift: the discipline has transitioned from a specialized niche of “alternative medicine” to a prominent focal point within integrative oncology (8). In this manuscript, we adopt the guideline-grounded definition of integrative medicine as “the coordinated use of evidence-based complementary practices and conventional care,” excluding approaches used in place of conventional treatment.

The annual output shows a clear transition from a relatively stable early period to accelerated growth after the mid-2010s. This inflection temporally coincides with the maturation of integrative oncology as a guideline-informed discipline, initially propelled by the evidence-based SIO clinical practice guideline by Greenlee et al. (9). In addition, the 2022 SIO–ASCO guideline on integrative medicine for pain management represents a contemporary benchmark of this paradigm shift, translating selected integrative modalities into actionable, indication-specific recommendations suitable for implementation in oncology settings (11). Such guidelines likely function as field-level “legitimizing events,” translating selected TCM-related interventions (e.g., acupuncture and mind–body therapies) from adjunctive practice into standardized supportive care options, thereby stimulating trial activity, evidence synthesis, and implementation-oriented research.

Geographically, the field exhibits a pronounced “bipolar” structure led by the United States and China, but their contributions appear complementary rather than redundant. The United States occupies a central position in collaboration networks and demonstrates high citation impact, consistent with its concentration of comprehensive cancer centers and established infrastructure for rigorous trial methodology and evidence-based medicine. Landmark institutions (e.g., MSKCC, MD Anderson) help anchor standardization—trial design, outcome measurement, and reporting quality—facilitating global uptake of integrative interventions as reproducible clinical protocols. In contrast, China contributes substantial publication volume, reflecting strong clinical accessibility, extensive patient throughput, and deep roots in TCM theory and practice (e.g., syndrome differentiation) (25, 26). However, the field would benefit from narrowing a persistent translational gap: heterogeneity in intervention protocols and variable adherence to international reporting standards can limit comparability and downstream guideline incorporation (27).

Taken together, these patterns argue for “integration by design,” not competition. Future progress will depend on combining Western methodological rigor with Eastern clinical breadth and therapeutic heritage (28). Strategic cross-continental collaborations—often bridged by internationally connected hubs (e.g., Australia and major US academic networks)—could enable multicenter RCTs with harmonized outcomes, transparent reporting, and culturally authentic intervention delivery, strengthening both external validity and real-world implementability (29).

### Bibliometric prominence versus clinical evidence grade

A central clinically relevant observation from our bibliometric mapping is a divergence between bibliometric prominence (publication volume, keyword dominance, and co-citation centrality) and guideline-based evidence strength. In our keyword network, acupuncture is the dominant modality (356 occurrences), whereas meditation (20 occurrences) and mindfulness (14 occurrences)—modalities that receive higher-grade recommendations for psychosocial outcomes in the SIO breast cancer guideline—are comparatively less visible. This inversion should not be interpreted as inconsistency in clinical efficacy, but rather as a field-level diagnostic signal: bibliometric dominance captures what is most frequently studied and cited within the retrieval boundary, whereas evidence grades reflect the consistency, methodological quality, and clinical relevance of outcomes across trials.

To make this distinction explicit, we added **Supplementary Table 4** to juxtapose modality prominence with key clinical evidence grades. Several factors may contribute to the observed divergence. First, our search strategy intentionally focused on TCM-labeled modalities (e.g., acupuncture, acupressure, Tai Chi/Qigong and other external therapies), whereas meditation/MBSR/relaxation are frequently indexed and conceptualized in behavioral medicine and psycho-oncology rather than within “TCM”, which may reduce their representation in a TCM-focused corpus. Second, acupuncture is widely accessible and culturally established in many settings, which facilitates trial production; however, across indications, acupuncture trials often vary in needling protocols, sham comparators, and sample size, which can constrain evidence grades despite high publication volume. Recognizing and reporting this prominence–evidence divergence enhances the clinical interpretability of bibliometric findings and prevents over-reading publication counts as efficacy strength.

### Clinical contextualization of core TCM modalities in survivorship care

Keyword clustering highlights a clinically coherent, symptom-oriented research architecture. The prominence of “Tai Chi” and symptom clusters such as “hot flashes” reflects a broader paradigm shift in oncology: as survival improves, care priorities increasingly focus on “living well,” addressing chronic sequelae of treatment rather than survival alone (30, 31).

Acupuncture is the bibliometrically dominant modality in this field (356 keyword occurrences) and anchors multiple keyword clusters related to endocrine therapy–associated symptoms, pain, and evidence synthesis (32). Importantly, publication volume should be distinguished from evidence quality and recommendation grade. In the SIO breast cancer guideline (9, 10), the strength of recommendation for acupuncture varies by indication; for several symptom domains, evidence was judged limited or heterogeneous, resulting in Grade C recommendations despite substantial research attention.

However, the evidence base for specific indications has matured significantly since 2017. Notably, a definitive multicenter randomized trial by Hershman et al. (3) demonstrated statistically significant improvement of AI-related joint pain with true acupuncture compared with sham and waitlist controls, supporting a more indication-specific evidence upgrade for aromatase inhibitor–associated musculoskeletal symptoms (AIMSS). For cancer pain, an updated synthesis reported that acupuncture and/or acupressure was associated with reduced pain intensity and decreased analgesic use, albeit with substantial heterogeneity, leading to moderate certainty of evidence (12). These findings align with the 2022 SIO–ASCO guideline on integrative approaches to pain management, which recommends acupuncture for AI-related joint pain in breast cancer and provides context-specific recommendations for other cancer pain syndromes (11).

Clinically, acupuncture is not a monolithic intervention. Our co-citation network includes multiple electroacupuncture trials, yet keyword aggregation may under-separate electroacupuncture from manual acupuncture. Because electroacupuncture and manual acupuncture differ in stimulation parameters, protocol standardization, and mechanistic hypotheses, future evidence synthesis and bibliometric work would benefit from distinguishing these subtypes and reporting key protocol elements (e.g., point selection, dosing/session frequency, comparator/sham design, and core outcomes). Overall, the field’s next step is likely not further expansion in trial count alone, but improved standardization, adequately powered multicenter designs, and harmonized endpoints to translate bibliometric centrality into higher-grade clinical confidence.

In stark contrast to acupuncture, acupressure occupies a smaller, more peripheral space in our keyword network, despite boasting comparatively clearer guideline support. The 2017 SIO guidelines recommend acupressure for mitigating chemotherapy-induced nausea and vomiting due to its favorable benefit-to-harm profile and practical feasibility for self- or caregiver-directed implementation (9). Moreover, the 2022 SIO–ASCO guidelines endorse acupressure for selected cancer pain contexts, emphasizing its scalability and accessibility (11). This striking divergence—between high clinical guideline readiness and modest bibliometric visibility—highlights a critical opportunity for future research to prioritize pragmatic effectiveness, implementation science, and the standardized reporting of dosing, fidelity, and patient-reported outcomes.

In parallel, Tai Chi and Qigong have established a significant presence as mind-body interventions. Unlike acupuncture, which is often utilized for targeted symptomatic relief, Tai Chi is conceptualized as a holistic practice (33). Bibliometric data reveal a specialized research focus for these modalities, primarily directed toward restoring upper-limb function post-surgery, reducing cancer-related fatigue, and alleviating psychological distress, including anxiety and depression (34). By integrating physical exercise with meditative focus, Tai Chi offers multimodal benefits in holistic rehabilitation, addressing the complex bio-psycho-social needs of survivors (35). Collectively, these findings indicate that the discipline has matured into a “symptom-oriented” research framework, where specific TCM modalities are meticulously aligned with distinct survivorship challenges to achieve a precision integrative oncology approach.

A small cluster labeled “Cancer of Unknown Primary” likely reflects cross-disease citation patterns rather than a true thematic shift away from breast cancer. This likely reflects the “cross-over” citation of high-quality RCTs that, while centered on general integrative care for advanced cancers, frequently include breast cancer subgroups or utilize breast cancer as a primary research model. Thus, this cluster does not signify a shift in subject matter, but rather underscores the interdisciplinary commonality of TCM interventions in addressing universal survivorship challenges, such as cancer-related fatigue and chronic pain (36).

### Evolution from clinical efficacy to implementation-oriented research

Establishing clinical efficacy is the foundation for integrating non-pharmacological TCM therapies into oncology. However, our trend-topic analysis indicates that the field has progressed through three strategic shifts. First, early research concentrated on managing discrete treatment-related side effects, with hotspots such as “hot flushes,” “tamoxifen,” and “vomiting” suggesting that TCM was initially positioned mainly as an adjunct to reduce the toxicity burden of chemotherapy and endocrine therapy (37, 38). By the mid-2010s, this emphasis broadened toward holistic survivorship, as the rise of terms like “quality of life,” “fatigue,” and “pain” reflects a transition from addressing isolated “symptoms” to supporting the “whole patient.” (39, 40) Second, the conceptual framing matured from “alternative medicine” to “integrative oncology,” a shift that goes beyond semantics and signals the gradual normalization of these approaches within mainstream supportive care; while acupuncture remains the central modality, the growing presence of mind–body exercises such as “Tai Chi,” “Qigong,” and “Yoga” demonstrates modal diversification and the formation of a multi-component intervention framework for breast cancer recovery (41–43). Third, the period from 2023 to 2025 highlights an emerging implementation- and precision-oriented phase, characterized by a surge in evidence-synthesis and translational keywords such as “network meta-analysis,” “scoping review,” and “feasibility,” implying that research priorities are moving from whether these therapies work to how they can be standardized, delivered, and scaled in real-world settings (44, 45). Concurrently, the surging prominence of hotspots like “sleep disturbance” and “insomnia” signals a refined research focus on the psychoneurological needs of cancer survivors (46). Importantly, this cluster is anchored by rigorous mind–body RCTs in breast cancer survivors, including the noninferiority trial demonstrating that Tai Chi Chih was statistically noninferior to CBT-I for insomnia outcomes with durable follow-up (47). Mechanistically, the concurrent emergence of “inflammation” aligns with subsequent biomarker and transcriptomic analyses showing that insomnia treatment—particularly Tai Chi—can reverse systemic, cellular, and genomic markers of inflammation in breast cancer survivors with insomnia (48), providing a plausible biological bridge between behavioral interventions and symptom clusters.

### Evolution from clinical efficacy to mechanistic exploration

While establishing clinical efficacy is fundamental to medical acceptability, elucidating the underlying biological pathways is essential for the scientific validation and global integration of TCM (49). Looking forward, the emergence of keywords such as “triple-negative breast cancer” and “apoptosis” hints at a long-term trajectory toward Precision Supportive Care. However, it is crucial to emphasize that the appearance of these subtype-specific terms currently reflects exploratory, hypothesis-generating mechanistic studies rather than guideline-ready clinical stratification. At present, there are no guideline-level recommendations tailoring TCM non-pharmacological interventions to specific molecular subtypes in breast cancer supportive care. Therefore, while future research may eventually prioritize subtype-specific strategies, biomarker-driven trials, and targeted nursing-led delivery models (48), these topics must currently be interpreted as an early research frontier with uncertain near-term clinical implications.

The frequency of mechanistic terms such as “inflammation” and “cells” has been rising. This shift indicates that the field is gradually entering a sophisticated phase of mechanistic inquiry (50, 51). The prominence of the keyword “inflammation” indicates a coordinated research initiative to examine how modalities like Tai Chi or acupuncture attenuate pro-inflammatory cytokines (e.g., IL-6, TNF-α) to mitigate cancer-related fatigue and cognitive deficits (34, 52). Similarly, the focus on “sleep disturbance” bridges behavioral interventions with neurobiological plasticity, potentially through the regulation of circadian rhythms and melatonin secretion (53, 54). This mechanistic trend is pivotal; it not only provides a biological rationale for symptom management but also paves the way for “Precision Rehabilitation.” By identifying specific biomarkers, such as inflammatory phenotypes, future TCM therapies can be tailored to patient subgroups most likely to derive benefit, thereby facilitating the seamless incorporation of traditional wisdom into the precision oncology framework (4, 43).

### Limitations

Several limitations should be considered. First, although our primary analyses used a merged WoSCC–Scopus corpus, coverage bias remains inevitable: relevant publications indexed only in other databases (e.g., CNKI and other regional repositories) were not included, which may particularly underrepresent Chinese-language clinical reports. Second, restricting the dataset to English-language articles and review articles introduces linguistic and document-type bias and may understate contributions from non-English scholarship or clinical practice–oriented formats. Third, bibliometric indicators are citation-dependent and therefore subject to time-lag; recent high-quality studies (especially 2023–2025) may be under-cited and should be interpreted as emerging frontiers rather than fully established hotspots. Finally, deduplication and cross-database harmonization may leave residual metadata inconsistencies (e.g., author name variants), despite systematic cleaning procedures. Additionally, although we screened for retracted publications and excluded confirmed retracted records prior to the final analyses, retraction status can change over time as databases are updated. Therefore, a small risk of future reclassification cannot be completely eliminated.

## Conclusion

This bibliometric analysis summarizes global research on non-pharmacological TCM-related interventions for breast cancer from 2000 to 2025, showing a marked acceleration in publications after the mid-2010s and a shift from “CAM” toward a more structured integrative oncology field. The research landscape is characterized by a US–China bipolar pattern: the United States contributes high-impact, methodologically rigorous evidence, whereas China provides substantial output supported by rich clinical resources, yet direct cross-regional collaboration remains limited. The thematic focus is predominantly survivorship- and symptom-oriented, with acupuncture and mind–body exercise (e.g., Tai Chi/Qigong) most frequently linked to quality-of-life outcomes such as pain, fatigue, hot flashes, mood, and sleep. Future work should emphasize multicenter, standardized trials and stronger international partnerships, alongside implementation- and mechanism-informed research, to support evidence-based and scalable integration into survivorship care.

## Data Availability

The original contributions presented in the study are included in the article. Further inquiries can be directed to the corresponding author.
