# Supplementary material for: Global research trends of TCM-based nonpharmacologic interventions in breast cancer supportive care: a bibliometric analysis (2000–2025)
Source: Front Oncol. 2026 Jun 10;16:1805786. doi: 10.3389/fonc.2026.1805786 (PMC13290594; doi:10.3389/fonc.2026.1805786)
Supplement: Supplementary file 1 [file Table1.docx]

**Supplementary**

doc S1.

Scopus：

TITLE-ABS-KEY ( "breast cancer" OR "breast cancers" OR "mammary cancer" OR "mammary cancers" OR "breast neoplasm" OR "breast neoplasms" OR "breast carcinoma" OR "breast carcinomas" OR "breast tumor" OR "breast tumors" OR "mammary neoplasm" OR "mammary neoplasms" OR "breast tumour")

AND TITLE-ABS-KEY ( "TCM non-pharmacological" OR "Traditional Chinese Medicine non-pharmacological" OR "TCM external therapy" OR "TCM external therapies" OR "Traditional Chinese Medicine external therapy" OR "acupuncture" OR "acupunctural" OR "electroacupuncture" OR "electro-acupuncture" OR "laser acupuncture" OR "warm needling" OR "warm needle" OR "fire needling" OR "fire needle" OR "plum blossom needle" OR "seven star needle" OR "intradermal needle" OR "thumbtack needle" OR "acupotomy" OR "needle knife" OR "miniscalpel needle" OR "acupressure" OR "shiatsu" OR "transcutaneous electrical acupoint stimulation" OR "tuina" OR "tui na" OR "anmo" OR "chinese massage" OR "moxibustion" OR "moxa" OR "auriculotherapy" OR "auricular therapy" OR "ear seed" OR "ear seeds" OR "vaccaria" OR "auricular acupressure" OR "auricular acupuncture" OR "ear acupressure" OR "ear acupuncture" OR "cupping therapy" OR "wet cupping" OR "dry cupping" OR "fire cupping" OR "moving cupping" OR "bleeding cupping" OR "bloodletting" OR "collateral pricking" OR "gua sha" OR "guasha" OR "catgut embedding" OR "thread embedding" OR "catgut implantation" OR "acupoint embedding" OR "acupoint implantation" OR "acupoint application" OR "acupoint sticking" OR "herbal ironing" OR "acupoint patch" OR "acupoint plaster" OR "herbal patch" OR "herbal plaster" OR "tianjiu" OR "sanfu" OR "san fu" OR "tai chi" OR "taichi" OR "taijiquan" OR "qigong" OR "qi gong" OR "daoyin" OR "baduanjin" OR "yijinjing" OR "wuqinxi" OR "liuzijue" )

AND NOT TITLE-ABS-KEY ( "green tea" OR "black tea" OR "coffee" OR "caffeine")

Wos：

#1: TS = ("breast cancer*" OR "mammary cancer*" OR "breast neoplasm*" OR "breast carcinoma*" OR "mammary neoplasm*" OR "mammary carcinoma*") OR (breast NEAR/2 (cancer* OR neoplasm* OR tumor* OR tumour* OR carcinoma* OR malignan*))

#2: TS = ("TCM non-pharmacological" OR "Traditional Chinese Medicine non-pharmacological" OR "TCM external therap*" OR "Traditional Chinese Medicine external therap*" OR acupuncture* OR electroacupuncture OR "electro-acupuncture" OR "laser acupuncture" OR "warm needling" OR "warm-needling" OR "warm needle" OR "needle warming moxibustion" OR "fire needling" OR "fire needle" OR "plum blossom needle" OR "seven-star needle" OR "intradermal needle" OR "thumbtack needle" OR acupotomy OR "needle-knife" OR needleknife OR "miniscalpel needle" OR acupressure* OR shiatsu OR "transcutaneous electrical acupoint stimulation" OR tuina OR "tui na" OR anmo OR ("chinese" NEAR/2 massage) OR moxibustion OR moxa OR auriculotherapy OR "auricular therapy" OR ("auricular" NEAR/2 (acupressure OR acupuncture OR point* OR acupoint*)) OR ("ear" NEAR/2 (seed* OR acupressure OR acupuncture OR point*)) OR "ear seed*" OR "ear-seed*" OR vaccaria OR "vaccaria seed*" OR "cupping therap*" OR "wet cupping" OR "dry cupping" OR "fire cupping" OR "moving cupping" OR "bleeding cupping" OR bloodletting OR "blood-letting" OR "collateral pricking" OR "gua sha" OR guasha OR "catgut embedding" OR "thread embedding" OR "catgut implantation" OR (acupoint* NEAR/3 (embedding OR implantation)) OR "acupoint application" OR "acupoint sticking" OR "herbal ironing" OR (acupoint* NEAR/3 (patch* OR plaster* OR sticking OR application)) OR (("herbal patch*" OR "herbal plaster*" OR cataplasm*) NEAR/5 acupoint*) OR tianjiu OR sanfu OR "san fu" OR "san-fu" OR "tai chi" OR taichi OR "tai ji" OR taijiquan OR qigong OR "qi gong" OR daoyin OR baduanjin OR "ba duan jin" OR "eight-section brocade" OR yijinjing OR "yi jin jing" OR wuqinxi OR "five-animal*" OR liuzijue OR "six healing sounds")

#3: TS = ("green tea" OR "black tea" OR "coffee" OR "caffeine")

#1 AND #2 AND NOT #3

Pubmed：

( "Breast Neoplasms"[Mesh] OR breast cancer[tiab] OR breast neoplasm*[tiab] OR mammary cancer[tiab] OR mammary neoplasm*[tiab] OR breast carcinoma[tiab] ) AND ( "Acupuncture Therapy"[Mesh] OR "Moxibustion"[Mesh] OR "Acupressure"[Mesh] OR "Tai Ji"[Mesh] OR "Qigong"[Mesh] OR "Auriculotherapy"[Mesh] OR "Cupping Therapy"[Mesh] OR "Bloodletting"[Mesh] OR acupuncture[tiab] OR electroacupuncture[tiab] OR "electro-acupuncture"[tiab] OR "warm needling"[tiab] OR "fire needling"[tiab] OR tuina[tiab] OR "tui na"[tiab] OR anmo[tiab] OR "chinese massage"[tiab] OR moxibustion[tiab] OR moxa[tiab] OR auricular[tiab] OR "ear seed"[tiab] OR vaccaria[tiab] OR cupping[tiab] OR "gua sha"[tiab] OR guasha[tiab] OR "catgut embedding"[tiab] OR "thread embedding"[tiab] OR "acupoint application"[tiab] OR "herbal patch"[tiab] OR sanfu[tiab] OR tianjiu[tiab] OR "tai chi"[tiab] OR qigong[tiab] OR baduanjin[tiab] OR yijinjing[tiab] OR wuqinxi[tiab] OR daoyin[tiab] ) NOT ( "Tea"[Mesh] OR "Coffea"[Mesh] OR green tea[tiab] OR black tea[tiab] OR coffee[tiab] OR caffeine[tiab] )

AND ("2000/01/01"[dp] : "2025/12/31"[dp])

AND english[lang]

Table S1. List of excluded retracted records

| **No.** | **Title** | **First Author** | **Journal** | **DOI** | **Year** | **Database flag** | **Retraction notice year** |
| --- | --- | --- | --- | --- | --- | --- | --- |
| 1 | Moxibustion Enhances Chemotherapy of Breast Cancer by Affecting Tumor Microenvironment | Xue, N | Cancer Management and Research | 10.2147/CMAR.S249797 | 2020 | WoSCC + PubMed | 2025 |
| 2 | The efficacy of Guolin-Qigong on the body-mind health of Chinese women with breast cancer: a randomized controlled trial | Liu, P | Quality of Life Research | 10.1007/s11136-017-1576-7 | 2017 | WoSCC + PubMed | 2017 |
| 3 | Effect of Chinese Traditional Wushu on Cancer-Related Fatigue, Sleep Quality and Upper Limb Dysfunction of Breast Cancer Survivors: A Systematic Review and Meta-Analysis | Ren, T | BioMed Research International | 10.1155/2022/6879566 | 2022 | WoSCC | 2022 |

Table S2. Year-by-year publication counts (2000–2025) in the main dataset (WOSCC-Scopus) and PubMed-derived counts.

| **Year** | **WOSCC-Scopus** | **PubMed** | **Year** | **WOSCC-Scopus** | **PubMed** |
| --- | --- | --- | --- | --- | --- |
| 2000 | 10 | 5 | 2013 | 69 | 27 |
| 2001 | 9 | 2 | 2014 | 59 | 22 |
| 2002 | 19 | 5 | 2015 | 62 | 29 |
| 2003 | 17 | 2 | 2016 | 71 | 34 |
| 2004 | 17 | 5 | 2017 | 73 | 27 |
| 2005 | 33 | 11 | 2018 | 93 | 36 |
| 2006 | 40 | 15 | 2019 | 89 | 33 |
| 2007 | 35 | 12 | 2020 | 107 | 38 |
| 2008 | 36 | 8 | 2021 | 131 | 49 |
| 2009 | 42 | 12 | 2022 | 140 | 48 |
| 2010 | 55 | 18 | 2023 | 139 | 43 |
| 2011 | 45 | 16 | 2024 | 137 | 50 |
| 2012 | 62 | 23 | 2025 | 171 | 66 |

Table S3. Top 20 major-topic MeSH terms and top 20 MeSH terms overall for the PubMed subset (N = 636).

| **Rank** | **Major MeSH** | **n** | **Percentage** | **All MeSH** | **n** | **Percentage** |
| --- | --- | --- | --- | --- | --- | --- |
| 1 | Breast Neoplasms | 393 | 77.21 | Humans | 504 | 79.25 |
| 2 | Acupuncture Therapy | 166 | 32.61 | Female | 461 | 72.48 |
| 3 | Antineoplastic Agents | 50 | 9.82 | Breast Neoplasms | 438 | 68.87 |
| 4 | Hot Flashes | 50 | 9.82 | Middle Aged | 217 | 34.12 |
| 5 | Cancer Survivors | 43 | 8.45 | Acupuncture Therapy | 203 | 31.92 |
| 6 | Tai Ji | 42 | 8.25 | Quality of Life | 160 | 25.16 |
| 7 | Complementary Therapies | 40 | 7.86 | Adult | 157 | 24.69 |
| 8 | Fatigue | 39 | 7.66 | Aged | 133 | 20.91 |
| 9 | Aromatase Inhibitors | 38 | 7.47 | Treatment Outcome | 115 | 18.08 |
| 10 | Neoplasms | 38 | 7.47 | Randomized Controlled Trials as Topic | 92 | 14.47 |
| 11 | Acupressure | 36 | 7.07 | Fatigue | 72 | 11.32 |
| 12 | Electroacupuncture | 34 | 6.68 | Hot Flashes | 67 | 10.53 |
| 13 | Quality of Life | 32 | 6.29 | Antineoplastic Agents | 63 | 9.91 |
| 14 | Arthralgia | 25 | 4.91 | Cancer Survivors | 54 | 8.49 |
| 15 | Survivors | 25 | 4.91 | Survivors | 52 | 8.18 |
| 16 | Qigong | 22 | 4.32 | Tai Ji | 49 | 7.7 |
| 17 | Nausea | 21 | 4.13 | Aromatase Inhibitors | 48 | 7.55 |
| 18 | Sleep Wake Disorders | 19 | 3.73 | Complementary Therapies | 48 | 7.55 |
| 19 | Menopause | 17 | 3.34 | Male | 44 | 6.92 |
| 20 | Vomiting | 16 | 3.14 | Acupressure | 38 | 5.97 |

Note: n indicates the number of records indexed with the term. “Major MeSH” refers to MeSH terms marked as a major topic in PubMed. “All MeSH” includes all assigned MeSH terms (including indexing/check tags).

Table S4 Comparison of bibliometric prominence and clinical evidence grades for core non-pharmacological interventions.

| **Intervention Modality** | **Bibliometric Frequency (n)** | **Clinical Indications** | **SIO Evidence Grade (2017)** |
| --- | --- | --- | --- |
| Acupuncture | 356 | Pain, fatigue, hot flashes, sleep disturbance, CINV | Grade C |
| Qigong | 57 | Quality of life, fatigue, sleep disturbance | Grade C |
| Tai Chi | 56 | Quality of life | Grade C |
| Acupressure | 44 | CINV | Grade B |
| Yoga | 36 | Anxiety/stress, depression/mood, quality of life | Grade B |
|  |  | Fatigue | Grade C |
| Electroacupuncture | 29 | CINV | Grade B |
| Meditation | 20 | Anxiety/stress, depression/mood, quality of life | Grade A |
| Massage | 17 | Anxiety/stress, depression/mood | Grade B |
|  |  | Pain | Grade C |
| Mindfulness | 14 | Anxiety/stress, depression/mood, quality of life | Grade A |

Note: Clinical indications and evidence grades strictly reflect the recommendations from the 2017 Society for Integrative Oncology (SIO) clinical practice guidelines for breast cancer. SIO Evidence Grades: Grade A = specific intervention is recommended; Grade B = specific intervention is recommended (with moderate certainty); Grade C = specific intervention is recommended with conditions (evidence is limited or heterogeneous). CINV: chemotherapy-induced nausea and vomiting.
